# Case Report: Primary Bilateral Minimally Invasive Adenocarcinoma of the Lungs in an 11-Year-Old Child: A Rare Case

**DOI:** 10.3389/fsurg.2021.741744

**Published:** 2021-10-26

**Authors:** Xing Lei, Yongfei Zheng, Guohua Zhang, Hailan Zheng

**Affiliations:** Department of Radiology, Taizhou First People's Hospital of Zhejiang Province, Taizhou, China

**Keywords:** lung tumor, minimally invasive adenocarcinoma, early lung adenocarcinoma, children, adolescents

## Abstract

There are many types of benign and malignant tissue, but primary lung tumor is very rare in children and often remains undiagnosed until after distant metastasis has occurred. Few cases of early lung adenocarcinoma in children have been reported. However, this case concerns an 11-year-old child with primary bilateral minimally invasive adenocarcinoma. As far as we know, this is the youngest reported case of its type.

## Case Presentation

The patient was an 11-year-old boy who visited the Taizhou First People's Hospital of the Zhejiang Province on June 13, 2019, having had a repeated cough and asthma symptoms for 30 days. The child underwent chest computed tomography (CT), and the results revealed two ground-glass opacities (GGOs) in the posterior segment of the left upper lobe apex and the anterior basal segment of the right lower lobe. At that time, the child was not treated. On November 15, 2020, a chest CT was performed in another hospital, which revealed that there were still GGOs in the posterior segment of the left upper lobe apex and the anterior basal segment of the right lower lobe.

**Figure 1:**
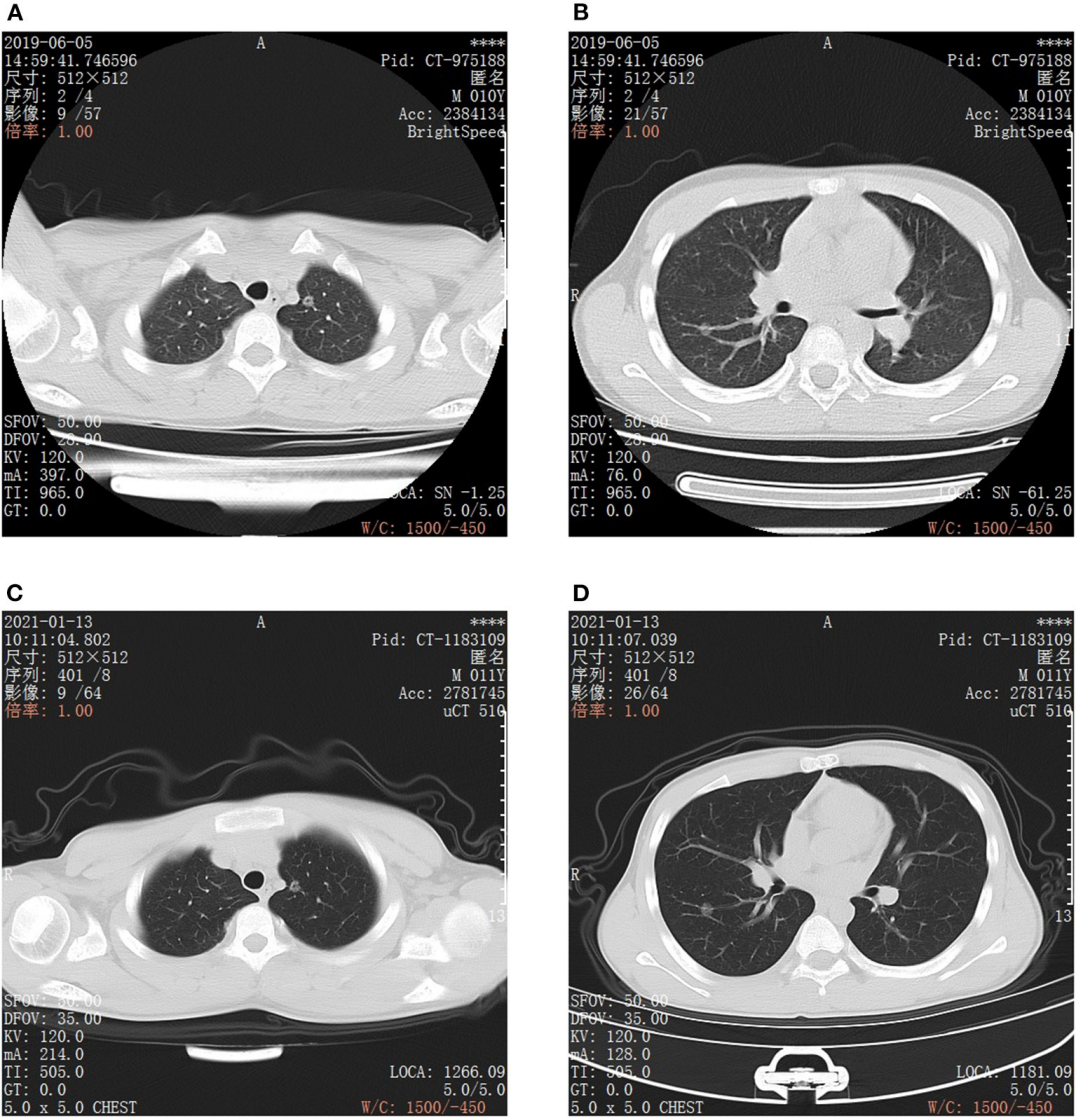
CT images of bilateral chest. **(A,B)** are CT scan images taken on June 5, 2019, Scanning model: GE BrightSpeed, scanning parameters: slice thickness: 5 mm, gap: 5 mm. **(A)** Round ground glass opacity in the posterior segment of the left superior lobe apex, the boundary is clear, in the center, there is a dot-like translucent shadow. **(B)** Round ground glass opacity in the anterior basal segment of the right lower lobe, the edge is smooth and clear, it can be seen that vascular shadow naturally passes through. **(C,D)** are CT scan images taken on January 13, 2021, scanning model: United image uCT, scanning parameters: slice thickness: 5 mm, gap: 5 mm. The 2 ground glass opacities of the posterior segment of the left upper lobe apex **(C)** and the anterior basal segment of the right lower lobe **(D)** are roughly similar to that of the film taken on June 05 2019. **(C)** CT image of ground glass opacity in the posterior segment of the left superior lobe apex. **(D)** CT image of ground glass opacity in the anterior basal segment of the right lower lobe.

On January 13, 2021, the child presented at our hospital for further treatment. After admission, he underwent a chest CT examination, which showed that there was no significant change in the GGOs when compared with imaging conducted in 2019 (see Figure 1). Laboratory examination showed that blood routine, liver and kidney function, carcinoembryonic antigen, alpha fetoprotein, carbohydrate antigen 19-9, carbohydrate antigen 72-4, cytokeratin 19 fragment, neuron specific enolase, serum ferritin, and other tumor markers were within the normal range. Ultrasonography of the liver, gallbladder, spleen, pancreas, and urinary system was negative, and no abnormality was found in a cranial MRI. The child was born after full-term, spontaneous labor, with no history of birth asphyxia rescue, previous health issues, lung or other tumors, blood transfusion, surgery, or infection. As the two lesions were most likely to be primary, early lung cancer, surgical resection was performed.

**Figure 2:**
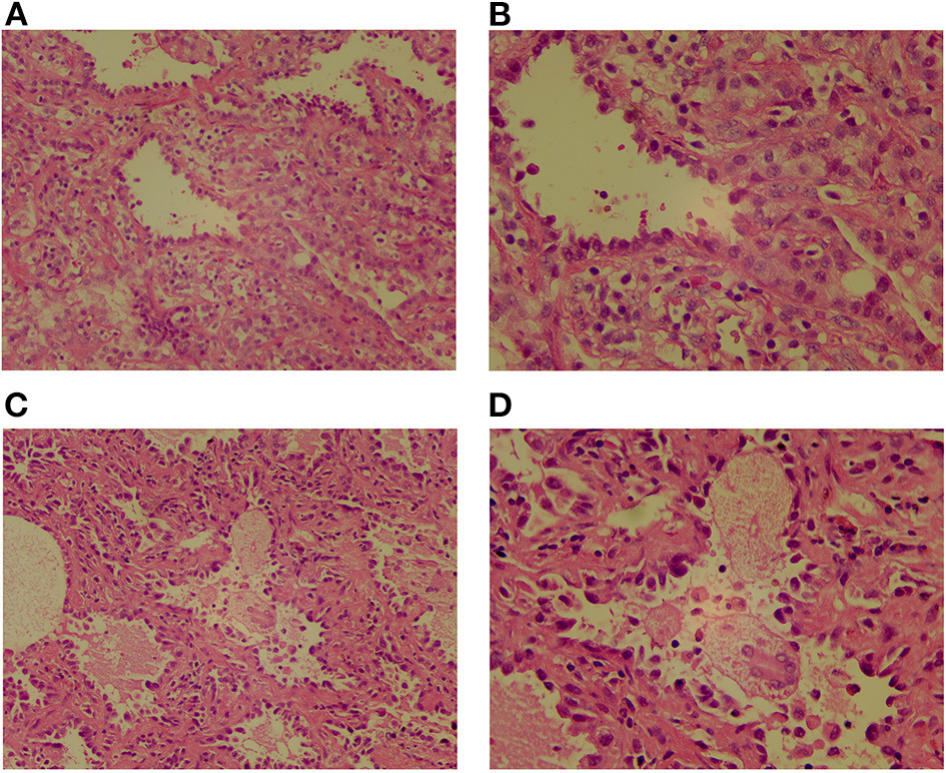
The pathological images of two GGOs show that the heteromorphic cells are arranged in the form of acini, the cells are dense, the nucleus is hyperchromatic, the cytoplasm is abundant and eosinophilic, peripheral fibrous tissue and lymphocyte proliferate. **(A)** Lesions in the posterior segment of the left upper lobe apex, HE ×200. **(B)** Lesions in the posterior segment of the left upper lobe apex, HE ×400. **(C)** Lesions in anterior basal segment of right lower lobe, HE ×200. **(D)** Lesions in anterior basal segment of right lower lobe, HE ×400.

**Figure 3:**
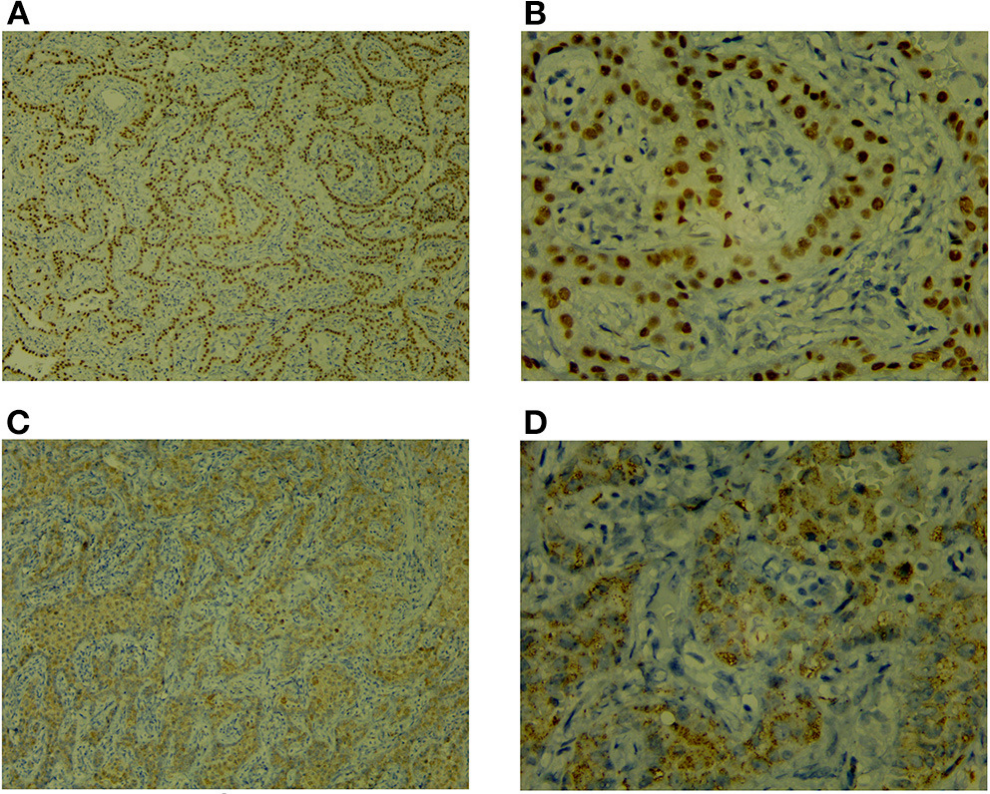
Immunohistochemistry [TTF-1 (+), Napsin A (+)]. **(A)** TTF-1 ×100. **(B)** TTF-1 ×400. **(A,B)** Positive staining of TTF-1 in the nuclei of lung adenocarcinoma. **(C)** NapsinA ×100. **(D)** NapsinA ×400. **(C,D)** Positive staining of NapsinA in the cytoplasm of lung adenocarcinoma.

The child underwent a video-assisted, thoracoscopic bilateral wedge resection, and the surgical pathological results revealed minimally invasive adenocarcinomas (MIAs) in the right lower lung and left upper lung, measuring 0.6 × 0.5 × 0.5 and 1.0 × 0.8 × 0.5 cm, respectively. No tumor thrombus was found in the vessel, and no cancer was found in the nerve bundle, but the lymph node tissue exhibited chronic inflammatory changes (group 10 lymph nodes and group 5/6 lymph nodes were sent for testing; see Figure 2). The immunohistochemical results were as follows: Ki-67 (5% +), Napsin A (+), thyroid transcription factor-1 (+), cytokeratin (CK) 5/6 (-), bronchial myoepithelium (P40 +), CK7 (+), EMA (+), vimentin (+), P63 (non-specific +; see Figure 3). A follow up on May 24, 2021 showed that the child had recovered well without any further discomfort.

## Discussion

The most common lung tumor types are carcinoid tumor, inflammatory myofibroblastic tumor, and pleuropulmonary blastoma, and the rare types include small cell carcinoma, adenocarcinoma, and pulmonary capillary angiomatosis, but primary lung tumors are very rare in children (1, 2), and the incidence is very low, estimated to be one per two million, accounting for 2% of all malignant tumors in children (3, 4). Like in adults, adenocarcinoma is the most common type of lung cancer in children, but the true incidence has not yet been determined (5, 6).

There is usually a delay between the clinical manifestations and the final diagnosis of lung cancer in children. Several studies listed non-specific symptoms, such as a persistent cough, and several courses of antibiotics being given to treat the patient before the cancer diagnosis was made. At present, only 6% of children with lung cancer seem to be asymptomatic (1, 5, 7–9). However, the non-specificity of the clinical manifestations and the rarity of the disease mean that many children have developed metastatic disease by the time of the diagnosis (10). Similarly, adults may also experience a delay in the diagnosis of lung cancer due to non-specific clinical manifestations (3).

Although the pathological features of adenocarcinoma in adults and children may differ to a certain extent, these characteristics have not yet been fully identified (11), and so the histopathological classification criteria of the subgroups of adult and child adenocarcinoma cases are the same at present.

In recent years, young women and non-smokers have shown an increased risk of lung cancer (12, 13). However, the risk of cancer in children and adolescents is rarely reported on. Recent literature (14) on clinical oncology, concerning eight 14–18-year-olds with early lung adenocarcinoma, seven of whom had MIA, stated that as is the case with adults, the early detection of lung adenocarcinoma in adolescents is accidental. The patients often exhibited no clinical symptoms, most of them had no family history of cancer, their imaging findings were mostly of small GGOs, pathology showed pre-invasive or invasive adenocarcinoma, the clinical stage was early (Ia), and the prognosis was good. In this study, the 11-year-old child had primary bilateral MIA, no history of smoking, no family history of cancer, and the clinical and imaging manifestations were consistent with the results of the aforementioned study.

MIA is a subtype of lung adenocarcinoma and is characterized by a small, solitary adenocarcinoma (<3 cm), with an infiltration range <5 mm. A study conducted by Yoshizawa et al. reported that epidermal growth factor receptor (EGFR) mutations were found in 83% of adult patients with MIA (15). At present, no specific carcinogenic factors or gene mutations have been found in children with lung cancer, although the incidence of some types of mutations varies with age (3). EGFR and other genes were not detected in this case.

The treatment of lung adenocarcinoma in children is consistent with that in adults (16), and surgery is an important positive prognostic factor in children with lung cancer (11). The 5-year survival rate of MIA after complete resection is 100%. At present, there is still a lack of consensus on the treatment of multiple GGOs (17). Zhang et al. noted that for suspected malignant sub-solid nodules and ipsilateral or contralateral GGOs with increased volume or solid components during follow-up, surgical resection is recommended (18). In this child, no solid components were found during the follow-up, but the volume had increased slightly (though not obviously).

Although no specific carcinogenic factors, gene mutations, tumor antigens, or other features tend to be found in children or adolescents with primary lung adenocarcinoma, most patients have metastatic disease by the time a diagnosis is made. Therefore, the chance for surgery is lost, the prognosis is very poor, the median survival time is 14 months, and the 5-year survival rate is 25% (1, 7).

In summary, the clinical symptoms of early lung adenocarcinoma are often nonspecific for both adults and children, and the disease is often only found by chance after a physical examination. Surgery is the first choice of treatment and has an excellent success rate.

## Data Availability Statement

The original contributions presented in the study are included in the article/supplementary material, further inquiries can be directed to the corresponding author/s.

## Author Contributions

HZ: conception, design of the research, and statistical analysis. XL and GZ: acquisition of data. YZ: analysis and interpretation of the data. XL: writing of the manuscript. HZ and XL: critical revision of the manuscript for intellectual content. All authors contributed to the article and approved the submitted version.

## Conflict of Interest

The authors declare that the research was conducted in the absence of any commercial or financial relationships that could be construed as a potential conflict of interest.

## Publisher's Note

All claims expressed in this article are solely those of the authors and do not necessarily represent those of their affiliated organizations, or those of the publisher, the editors and the reviewers. Any product that may be evaluated in this article, or claim that may be made by its manufacturer, is not guaranteed or endorsed by the publisher.
